# The Association Between Mindfulness and Athletes’ Distress Tolerance: The Mediating Roles of Cognitive Reappraisal and Mental Toughness

**DOI:** 10.3390/bs15030298

**Published:** 2025-03-03

**Authors:** Zhangyi Zhong, Hongyu Jiang, Huilin Wang, Yang Liu

**Affiliations:** 1 School of Physical Education, Hunan University of Science and Technology, Xiangtan 411201, China; 23011701021@mail.hnust.edu.cn (Z.Z.); 1170018@hnust.edu.cn (H.J.); 2 School of Business, Hunan University of Science and Technology, Xiangtan 411201, China; 1150141@hnust.edu.cn; 3 Faculty of Business Administration, University of Macau, Avenida da Universidade, Taipa, Macau, China

**Keywords:** mindfulness, cognitive reappraisal, mental toughness, distress tolerance, athletes

## Abstract

Physical and psychological distress frequently challenges athletes throughout their careers. The perception of pain and coping strategies are often crucial factors in achieving victory. These factors not only reflect their commitment to daily training, but can also indicate their level of athletic performance. This study is a cross-sectional research using convenience and snowball sampling methods. It explores the relationship between mindfulness and athletes’ distress tolerance, revealing the mediating roles of cognitive reappraisal and mental toughness. A sample of 285 athletes was drawn from universities, youth training centers, and sports academies in Hunan, Hubei, and Sichuan provinces in China. To assess the proposed hypotheses, structural equation modeling was conducted using AMOS v23. The findings identified a significant positive correlation between mindfulness, cognitive reappraisal, and mental toughness. Additionally, both cognitive reappraisal and mental toughness were positively associated with distress tolerance. Further analysis demonstrated that cognitive reappraisal and mental toughness function as mediators in the mindfulness–distress tolerance relationship. These results indicate that athletes with higher mindfulness levels exhibit enhanced cognitive reappraisal skills, greater mental toughness, and improved distress tolerance. This means that athletes with higher mindfulness levels are more likely to detach from negative psychological states in a timely manner, utilizing emotional regulation skills such as cognitive reappraisal, and face training and competition with greater mental resilience. This can help athletes alleviate negative psychological states and, to some extent, reduce their experience of pain, enabling them to better cope with challenges. Therefore, athletes can actively engage in mindfulness practices combined with cognitive reappraisal strategies to achieve better psychological states, which can support their adherence to training and rehabilitation plans.

## 1. Introduction

Every athlete experiences various forms of physical or psychological discomfort at different stages of their career. These discomforts often lead to emotions such as worry, anxiety, and anger. As the physical strain and negative emotions intensify, they can ultimately have a serious impact on the athlete’s physical and mental health (Mohammed et al., 2018). Enhancing athletes’ ability to tolerate negative physical and mental states can improve their overall well-being and increase the likelihood of achieving superior athletic performance (Moshier et al., 2013). Statistics indicate that approximately 3 to 17 million individuals suffer injuries annually in recreational and competitive sports (Bijur et al., 1995; Kraus & Conroy, 1984). Among elite team sports athletes, nearly 35% have experienced mental health issues (Beable et al., 2017).

According to Stephan and Brewer (2007), athletes primarily aim to secure success by engaging in rigorous and effective training sessions. However, physical injuries and emotional distress—whether temporary or permanent—pose significant emotional and cognitive challenges that hinder their training and competition efforts. The distress athletes face includes competition pressure, anxiety during training, frustration after failure, self-reproach (Bernstein et al., 2009; Carpenter et al., 2019), sports injuries, pain during rehabilitation, and irreversible structural changes in their bodies to adapt to their sports (Assa et al., 2019; Ryan & Kovacic, 1966). Athletes who remain in these negative psychological and physical states are at a much higher risk of engaging in extreme behaviors, such as suicide or self-harm, which can lead to the premature end of their careers (Baum, 2005; Rice et al., 2016).

An athlete’s capacity to manage distress is essential for maintaining overall physical and mental well-being. It significantly influences the effectiveness of their training and recovery efforts, ultimately shaping the trajectory of their athletic career (Rice et al., 2016). Throughout their career, pain and injuries are almost inevitable. How athletes perceive and manage pain, as well as the strategies they use to mitigate it, demonstrate their dedication to training and impact the effectiveness of rehabilitation. These factors significantly affect their physical fitness, performance outcomes, and competitive achievements (Maffulli et al., 2010). Research by Pen and Fisher (1994) revealed that most athletes who suffer acute injuries during training or competition do not receive timely treatment, often worsening the condition into chronic injuries (Timpka et al., 2014). Recovering from chronic injuries often entails enduring significant pain and is essential for refining an athlete’s technical and tactical skills, ultimately influencing their performance results. Some athletes, unable to endure the chronic rehabilitation process, even abandon their athletic careers (Christakou & Lavallee, 2009). Additionally, Ryan and Kovacic (1966) observed a connection between an athlete’s capacity to endure physical pain and the nature of their sport. In physically demanding disciplines such as football and rugby, the ability to tolerate distress is often a critical determinant of success. In sports like tennis and golf, while distress tolerance may not be the decisive factor for victory, it can help athletes better withstand negative emotions and external factors, allowing them to execute strategies more effectively and achieve better outcomes.

Beyond physical pain, athletes bear significant psychological burdens. Many elite athletes begin specialized training in childhood or adolescence, with many dedicating over a decade to their sport (Baker et al., 2003). This prolonged engagement often results in physical and psychological fatigue, leading to burnout and negative emotional states such as boredom (Schinke et al., 2018). When athletes face high-intensity training, immense pressure, or prolonged periods of inactivity, they frequently experience depression, anxiety, anger, diminished self-esteem, loss of identity, loneliness, and fear (Menke & Germany, 2019; Şenışık et al., 2021; Smith et al., 1993). Athletes face a variety of mental health challenges associated with their sport, such as collision-related concussions (Grady, 2010), reliance on performance-enhancing substances (Barnes et al., 2022), exercise-related eating disorders like anorexia (Sudi et al., 2004), and compulsive exercise patterns (Li et al., 2024). Mohammed et al. (2018) demonstrated that athletes with higher distress tolerance can better manage mental health challenges, effectively regulate their emotions, and reduce adverse consequences, ultimately improving performance while safeguarding their physical and mental health.

In conclusion, distress tolerance is vital for maintaining athletes’ physical and mental well-being, as well as enhancing their performance. While previous studies have largely concentrated on assessing distress tolerance (Garner et al., 2018; Simons & Gaher, 2005), its effects on health outcomes (Castaldelli-Maia et al., 2019; Leyro et al., 2010), and athletic performance (Moshier et al., 2013; Ryan & Kovacic, 1966), there remains limited exploration of the cognitive mechanisms contributing to distress tolerance. Whitmarsh and Alderman (1993) found that acquiring practical sports skills and engaging in stress inoculation training (SIT) can help athletes reduce physical discomfort. Similarly, Mohammed et al. (2018) emphasized that mindfulness-based stress management techniques enhance pain tolerance in injured athletes, supporting more efficient rehabilitation.

Building on this, the study aimed to (1) identify the primary factors leading to distress among athletes; (2) analyze the correlations between mindfulness, cognitive reappraisal, mental toughness, and distress tolerance, and examine the mediating roles of cognitive reappraisal and mental toughness; and (3) provide practical strategies to alleviate athletes’ pain experiences, improve their tolerance of physical and psychological discomfort during competitions, and achieve better performance outcomes. This study, while analyzing the causes of discomfort among athletes, also explores the connection between mindfulness and distress tolerance, as well as the mediating roles of cognitive reappraisal and mental toughness. It proposes mindfulness meditation as a strategy to bolster cognitive reappraisal skills and mental toughness, both of which are critical to enhancing distress tolerance during training and competitions. By addressing these psychological dimensions, this study contributes not only to advancing theoretical insights in the field, but also to delivering practical guidance for tailored interventions. Additionally, the findings of this study can help athletes enhance their physical and mental adaptability, improve the rehabilitation outcomes of sports injuries, and display better athletic performance in competitions, leading to superior results.

## 2. Literature Review and Hypothesis Development

### 2.1. The Concept of Variables

#### 2.1.1. Mindfulness

Mindfulness, originating from Buddhist traditions, traces its roots to the ancient Sanskrit term “Sati”, which emphasizes awareness and presence in the moment. With a history spanning over 2500 years, mindfulness is recognized in psychology as a multifaceted practice involving self-awareness, focused attention, and memory (Siegel et al., 2009). Traditionally, it has been defined as the sustained self-observation of changes in emotional, physical, and environmental states in the present moment. Furthermore, mindfulness is often described as a mental state cultivated through practices such as meditation (Kabat-Zinn, 2003).

Over time, mindfulness has transitioned from its spiritual roots to become a widely adopted approach in psychology and healthcare. It is primarily understood as the cultivation of present-focused awareness, characterized by calmness, acceptance, and compassion (Baer, 2011). As research on mindfulness has flourished, its applications have extended to various domains, including pain management, mental health enhancement, and supportive care for individuals undergoing cancer treatment (Melbourne Academic Mindfulness Interest Group, 2006).

In current research, mindfulness is commonly divided into two categories: state mindfulness and trait mindfulness. State mindfulness reflects the mindfulness experienced in a specific moment, while trait mindfulness represents an individual’s consistent tendency to maintain mindfulness across various contexts and over time. Although these two constructs are closely related, they are conceptually distinct (Sala et al., 2020). Given that most theoretical frameworks and empirical studies emphasize trait mindfulness in relation to health behaviors, this study adopts a trait mindfulness perspective.

In the realm of sports, mindfulness is commonly described as a state of focused and non-judgmental awareness that enables athletes to engage fully with their physical and mental performance (Bernier et al., 2009). Studies indicate that mindfulness practices, such as meditation, can improve athletes’ concentration, help them accept mistakes without harsh self-criticism, enhance emotional regulation, and manage negative emotions like anger, fear, and tension more effectively. These benefits collectively contribute to optimizing athletic performance (Birrer et al., 2012; Carmody et al., 2009).

#### 2.1.2. Distress Tolerance

In the fields of psychopathology, clinical medicine, and psychology, distress tolerance remains a central topic of investigation for researchers. It is defined as an individual’s ability to manage and endure both psychological and physical discomfort (Simons & Gaher, 2005; Woolf, 2010). This concept encompasses coping with negative emotional states as well as external physical challenges like pain. Within psychology and psychopathology, distress tolerance is regarded as a vital construct, serving as both a significant area of study and an essential psychological outcome. Research has shown that low distress tolerance is closely associated with various mental health issues, including anxiety disorders (Carleton et al., 2012), depression (Williams et al., 2013), and disordered eating behaviors (Anestis et al., 2007).

For athletes, the capacity to handle distress is particularly important. They are required to handle the high-pressure environment of competitive sports while also managing the constant evaluation and criticism from the public (Zhong et al., 2024). Moreover, the physical injuries and discomfort that often accompany daily training pose significant challenges to their overall well-being. Athletes with limited capacity for distress tolerance may struggle to maintain emotional balance, making them more vulnerable to mental health challenges and psychological strain.

During daily training, individuals with a higher distress tolerance can endure the physiological discomfort of muscle overextension, joint wear, and the monotony and anxiety of repetitive exercises, resulting in better training outcomes (Pinkerton et al., 1989). In competitive settings, however, muscle and joint pain, along with negative psychological states, can prevent athletes from performing at their best. Moreover, the discrepancy between their actual and expected performance can heighten negative emotions such as tension and anxiety. Athletes with weaker emotional regulation skills may even engage in impulsive behaviors with severe consequences, which not only significantly affects competition outcomes, but may also impact the entire trajectory of their careers (Pen & Fisher, 1994).

#### 2.1.3. Cognitive Reappraisal

Cognitive reappraisal refers to the process of reinterpreting a situation or stimulus to alter its emotional significance and reduce its impact (Gross, 1998). This emotion regulation strategy acts early in the emotional process, addressing potential emotional responses before they fully develop. By reshaping the way events are perceived, cognitive reappraisal can redirect emotional trajectories and lessen the impact of emotional reactions that might otherwise occur (Gross & John, 2003).

A variety of behavioral studies have demonstrated the effectiveness of cognitive reappraisal in promoting emotional regulation and improving mental health. Its advantages span diverse domains, including physiological changes (Ray et al., 2010), neural responses linked to emotional processing (Buhle et al., 2014), self-reported emotional experiences (Brockman et al., 2017; Gross, 1998), and decision-making processes influenced by emotional states in economic contexts (McRae et al., 2012; van’t Wout et al., 2010).

For athletes, the pressure they face before and during competitions often leads to emotional dysregulation (Guyon et al., 2020; Milne & Neely, 2022). Cognitive reappraisal can serve as a timely intervention to address negative emotions before they adversely affect athletic performance, thereby contributing to better competition outcomes (Robazza et al., 2023). For instance, when facing a formidable opponent, an athlete might reframe the fear and perceived threat of the competitor into a challenge and an opportunity for growth. Viewing the situation as a chance to surpass personal limits and gain valuable experience can help prevent the negative effects of maladaptive cognitions (Robazza et al., 2008), ultimately leading to both competitive success and personal development.

#### 2.1.4. Mental Toughness

In the field of sports psychology, mental toughness is frequently regarded as a subject of considerable interest and debate. Despite this attention, it remains one of the most challenging and least understood constructs within the discipline (Crust, 2007). The term was first introduced by Cattell (1957), who described mental toughness as a realistic, self-reliant behavior representing a resilient, masculine personality dimension. Research on athletes’ mental toughness began with applied sports psychologist Loehr (1982), who suggested that mentally tough athletes respond to negative emotions differently, allowing them to stay relaxed, calm, and energized.

Subsequent studies on athletes’ mental toughness have proliferated, but varying definitions and the resulting operational approaches have added complexity and confusion to this concept (G. Jones et al., 2002). To clarify this, the current study utilizes the definition of mental toughness proposed by G. Jones et al. (2002). This definition describes mental toughness as a psychological edge—whether innate or developed—that enables athletes to confront the demands of their sport, such as competition, training, and daily challenges, more effectively than their counterparts. The key aspects include maintaining consistency, demonstrating determination and confidence, sustaining focus, and managing pressure during competitions.

Research has highlighted several core traits of mental toughness, such as strong self-belief, resilience, optimism, and the ability to persist and maintain focus even in demanding situations (Thelwell et al., 2005). These attributes are recognized as crucial for enhancing athletic performance and achieving success in competitive settings (Wilson et al., 2019).

### 2.2. Hypotheses

#### 2.2.1. Mindfulness, Cognitive Reappraisal, and Mental Toughness

The environment around us constantly exerts various influences on individuals, both beneficial and harmful. On one hand, individuals experience joy and well-being from positive stimuli; on the other hand, they are daily exposed to numerous stresses and pressures, with their psychological state and emotional health being under constant threat from negative stimuli and stress (Rutter, 2005; Tost et al., 2015). Research has shown that mindfulness has a positive effect on individuals’ emotional health (Grossman et al., 2004), emotional regulation abilities (Hill & Updegraff, 2012), and even overall mental health (Gu et al., 2015). Although mindfulness cannot reduce the objective stress and stimuli individuals face, mindful individuals are able to focus on the present stress and stimuli in a non-judgmental manner. This allows them to detach from negative emotions, actively regulate their mood, and, to some extent, avoid the harm that such negative psychological states and emotional health may face (Chua et al., 2025; Hilton et al., 2017; Jin et al., 2020).

Additionally, in their foundational exploration of the Mindful Coping Model, E. Garland et al. (2009) identified mindfulness and positive cognitive reappraisal as distinct yet interconnected elements that collaboratively facilitate effective emotional regulation. While they operate independently, they also reinforce one another. Subsequently, E. L. Garland and Howard (2013) further investigated mindfulness meditation, suggesting that its practice enables individuals to detach from anxiety and stress, entering a non-evaluative state. In this state, automatic emotional reactions are interrupted, attention gradually broadens, and individuals become aware of previously overlooked details. Over time, as habitual responses change and new information is incorporated, individuals develop new perceptions of their environment or experiences, enhancing cognitive flexibility through mindfulness (E. L. Garland et al., 2015).

For athletes, whose performance is often influenced by emotions, high emotional control is essential for ensuring optimal performance (Laborde et al., 2016). Research also highlights a close relationship between mindfulness and mental toughness (M. I. Jones & Parker, 2018). Andersen (2012) proposed that focusing attention on the present during mindfulness meditation aligns with the attention regulation dimension of mental toughness, a finding consistent with Gucciardi et al. (2015). Josefsson et al. (2017) investigated how mindfulness functions within athletic settings and found that consistent mindfulness meditation practice prior to competitions helps athletes manage negative emotions and develop greater mental resilience. Similarly, research by Birrer et al. (2012) indicates that mindfulness supports the development of psychological skills, helping individuals cultivate a more positive and focused mindset when facing challenges.

Studies investigating the link between cognitive reappraisal and mental toughness have mainly emphasized its role in emotional regulation (Mutz et al., 2017) and stress reduction (Riepenhausen et al., 2022). Mutz et al. (2017) observed that individuals who frequently employ cognitive reappraisal exhibit greater mental toughness compared to those relying on strategies like expressive suppression. Similarly, Lin et al. (2017) identified a strong positive association between mental toughness and the regular use of cognitive reappraisal. Their findings suggest that individuals with higher mental toughness are more inclined to integrate cognitive reappraisal into their daily coping strategies.

In competitive sports, athletes who actively apply cognitive reappraisal are better equipped to handle external pressures, maintain constructive self-dialogue, and reinterpret challenges and emotional experiences in a more positive light. This mental strategy strengthens their resilience, ultimately leading to enhanced performance in competitions (Robazza et al., 2023). Based on this, the following hypotheses are proposed:

**Hypothesis 1.**  *There is a positive association between mindfulness and cognitive reappraisal, such that higher levels of mindfulness are associated with greater cognitive reappraisal.*

**Hypothesis 2.**  *There is a positive association between mindfulness and mental toughness, such that higher levels of mindfulness are associated with greater mental toughness.*

**Hypothesis 3.**  *There is a positive association between cognitive reappraisal and mental toughness, such that greater cognitive reappraisal is associated with higher levels of mental toughness.*

#### 2.2.2. Cognitive Reappraisal, Mental Toughness, and Distress Tolerance

Distress tolerance is defined as an individual’s capacity to endure challenging physical and psychological experiences. Research underscores a robust theoretical link between emotional regulation and distress tolerance (Leyro et al., 2010). Athletes often face muscle and joint injuries and pain during training and competition, while simultaneously being influenced by psychological factors such as pressure, tension, and fear. These objective physical pains and negative psychological states not only significantly impact their performance and outcomes, but also pose substantial harm to their physical and mental health (Mohammed et al., 2018). Robazza et al. (2023) proposed that when athletes are faced with significant physical strain and psychological distress, they should actively engage in emotional regulation to manage the physical and mental pressures, thereby improving both their performance and overall health. Gross (1998) characterized cognitive reappraisal as an early-stage emotion regulation approach that mitigates negative emotional reactions by reshaping how situations are interpreted. Cognitive reappraisal enables athletes to detach from their current emotional state, reframe the challenges they face, and adopt a fresh, more positive mindset to approach the upcoming competition, thus reducing anxiety and other painful emotions (Gross, 2002).

Moreover, the self-efficacy theory suggests that emotions are a key source of self-efficacy, and engaging in positive cognitive reappraisal can enhance athletes’ emotional experiences, boosting their perception of personal ability and increasing their tolerance for painful experiences (Morina et al., 2018; Wang et al., 2022). Similarly, Feldner et al. (2003) observed that while individuals with higher cognitive reappraisal skills encounter the same levels of psychological distress and physical pain as others, they report feeling significantly less subjective discomfort. Additionally, cognitive reappraisal has been recognized as particularly effective within emotion regulation strategies, especially for improving distress tolerance (Wolgast et al., 2011).

While the external stimuli and injuries an individual endures are relatively objective and difficult to alter, the experience of distress is subjective. People’s perception of suffering varies depending on their environment, emotions, and psychological state (M. I. Jones & Parker, 2018). Mental toughness, a complex psychological attribute, is identified as critical for managing high-pressure situations (Connaughton et al., 2008). According to G. Jones et al. (2002), athletes with heightened mental toughness can overcome physical and emotional pain barriers, sustaining exceptional performance and consistent effort in competitive scenarios. Furthermore, Levy et al. (2006) established a positive relationship between mental toughness and distress tolerance, indicating that athletes with greater mental toughness are better equipped to manage both psychological and physical challenges, enhancing resilience and performance (Birrer & Morgan, 2010).

Based on this, the following hypotheses are proposed:

**Hypothesis 4.**  *There is a positive association between cognitive reappraisal and distress tolerance, such that greater cognitive reappraisal is associated with higher distress tolerance.*

**Hypothesis 5.**  *There is a positive association between mental toughness and distress tolerance, such that higher mental toughness is associated with greater distress tolerance.*

#### 2.2.3. The Mediating Effects

Research on the mediating roles of cognitive reappraisal and mental toughness has largely concentrated on their connections with mental health issues, including depression (Mutz et al., 2017), anxiety (Goldin et al., 2012), and overall psychological well-being (Riepenhausen et al., 2022). At the same time, mindfulness has been extensively studied for its impact on emotional regulation (Chambers et al., 2009) and its ability to enhance mental toughness (Andersen, 2012). By fostering a present-focused mindset, mindfulness helps individuals distance themselves from negative emotions like stress and anxiety, facilitating emotional regulation and altering their perception of challenges (E. L. Garland et al., 2015). This enables them to better cope with pressure and remain engaged in their personal and professional lives (Bell et al., 2013).

Moreover, evidence from studies by Uphill and Dray (2009) and Doorley et al. (2022) indicates that employing positive cognitive reappraisal significantly strengthens mental toughness. By adopting strategies to regulate emotions, leveraging psychological tools, and cultivating resilience, individuals can shift their perspective on adversity, allowing them to face challenges with greater composure and confidence (Lin et al., 2017).

Based on this, the following hypothesis is proposed:

**Hypothesis 6.**  *Cognitive reappraisal and mental toughness sequentially mediate the association between mindfulness and distress tolerance, such that mindfulness is associated with greater cognitive reappraisal and mental toughness, which in turn enhances distress tolerance.*

**Figure 1:**
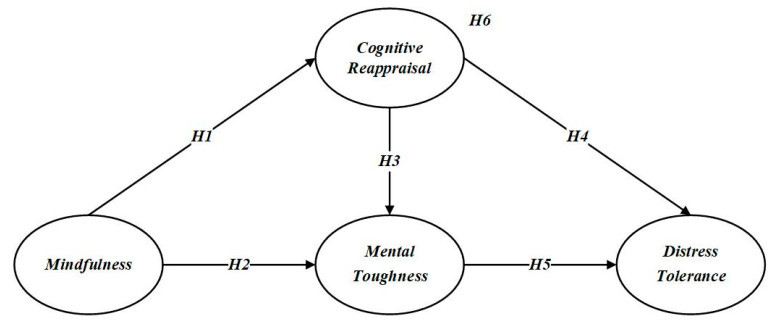
Hypothetical model.

A summary of all hypotheses is illustrated in Figure 1.

## 3. Methodology

### 3.1. Participants and Procedures

This study used snowball and convenience sampling techniques to recruit adolescent athletes aged 16–25 years with a sports level of second tier or above. In China, second-tier and higher athletes include second-tier athletes, first-tier athletes, and national-level athletes. Second-tier athletes are those who have qualified in provincial, autonomous region, or municipality sports championships or tournaments jointly organized by sports and education administrative departments. First-tier athletes are those who meet the performance standards in national comprehensive sports competitions or championships organized by provincial, regional, or municipal sports departments. National-level athletes are those who have met the performance standards in national sports competitions, including the National Games, National Youth Games, National Championships, National Youth Championships, National Junior Championships, National Indoor Championships, National Grand Prix, National Individual Events, and National Team Events, as well as the National Student Games and National Middle/High School Championships.

Data were collected during November and December 2024 through an online questionnaire distributed at youth training centers, universities, and sports schools in Hunan, Hubei, and Sichuan provinces. Recruitment efforts included sharing invitations via social media platforms such as WeChat and Weibo, encouraging potential participants to take part. Interested individuals received a link to the questionnaire, which outlined the study’s purpose and emphasized its voluntary nature. As a gesture of appreciation, participants were offered a reward valued at 10 CNY upon completing the survey. Additionally, they were encouraged to share the questionnaire with teammates and peers to broaden the participant pool.

A total of 334 questionnaires were distributed, resulting in 285 valid responses, yielding an effective response rate of approximately 85.3%. The demographic characteristics of the respondents are summarized in Table 1. Key observations include the following: (1) Gender distribution was nearly equal, with a male-to-female ratio of approximately 1:1. (2) About 70% of respondents were between the ages of 17 and 23. (3) Badminton and athletics were the most frequently reported sports, accounting for roughly one-third of the activities. (4) Half of the participants reported experiencing between 7 and 9 injuries in training or competition over the past year.

### 3.2. Instruments

The questionnaire for this study was structured into five distinct sections. The first section focused on demographic information, collecting details such as gender, age, type of sport, and the number of injuries sustained during training or competition in the past year. The second section examined mindfulness levels using the Mindful Attention Awareness Scale (MAAS), developed by Van Dam et al. (2010) which included five items. An example item was: “It seems I am ‘running on automatic’, without much awareness of what I’m doing”. The third section assessed distress tolerance through the Distress Tolerance Scale-Short Form (DTS-SF) by (Garner et al., 2018), comprising four items. A sample statement from this section was: “My feelings of distress are so intense that they completely take over”. Cognitive reappraisal was measured in the fourth section using the Emotion Regulation Questionnaire-Short Form (ERQ-S), created by Preece et al. (2023), which included six items. One example was: “When I want to feel more positive emotion (such as joy or amusement), I change the way I’m thinking about the situation”. The fifth and final section evaluated participants’ mental toughness using the Mental Toughness Scale (MTS) by Madrigal et al. (2013), which contained seven items. A representative item read: “When an obstacle is in my way, I find a way to overcome it”.

### 3.3. Data Analysis

This study used AMOS v23 to construct a structural equation model (SEM) to explore how mindfulness enhances athletes’ cognitive reappraisal and mental toughness, thereby improving distress tolerance. Model parameters were estimated using the maximum likelihood (ML) method, and a two-step approach was adopted. The first step assessed the reliability and validity of the measurement model, while the second examined the structural model’s fit indices, path coefficients, and mediation effects.

To address the potential common method variance (CMV) in self-reported data, the method proposed by Mossholder et al. (1998) was applied. A comparison of two models showed that Model 1 had a chi-square value of 618.155 (*df* = 203, *p* < 0.001), while Model 2 yielded 575.829 (*df* = 203, *p* < 0.001). These findings indicate that Model 1 provides an adequate fit and that CMV is not a significant concern in this study.

## 4. Results

### 4.1. Measurement Model

The researchers conducted confirmatory factor analysis (CFA) using AMOS v23 to evaluate the reliability and validity of the latent constructs. As shown in Table 2, all variables achieved a Cronbach’s α above 0.8, demonstrating strong internal consistency (Fornell & Larcker, 1981). Additionally, average variance extracted (AVE) values exceeded 0.6, and composite reliability (CR) estimates were above 0.8, confirming robust convergent validity. Factor loadings from principal component analysis ranged between 0.690 and 0.887, further supporting construct validity.

### 4.2. Structural Model

**Figure 2:**
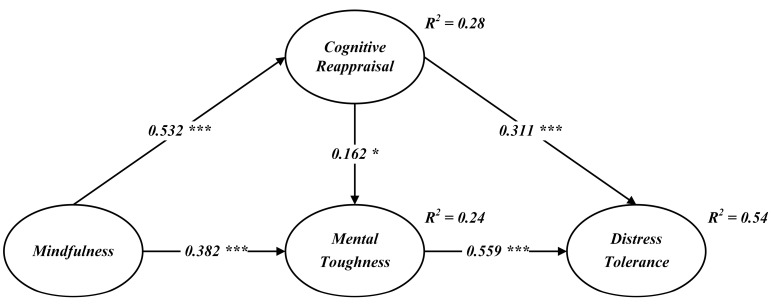
Structural path model. Note: * *p* < 0.05, *** *p* < 0.001.

After assessing the reliability and validity of the measurement model, the structural model was analyzed using AMOS v23 to evaluate the proposed hypotheses. The CFA, conducted with a bootstrap sample size of 5000, satisfied the standard fit criteria (χ²/df = 2.315, NFI = 0.904, TLI = 0.934, CFI = 0.943, RMSEA = 0.068), indicating a strong alignment between the model and the observed data. Pearson correlation analysis (Table 3) further verified significant relationships among the constructs, with standardized coefficients displayed in Figure 2.

As shown in Figure 2, mindfulness had a significant positive effect on both cognitive reappraisal (*β* = 0.532, *p* < 0.001) and mental toughness (*β* = 0.382, *p* < 0.001), supporting H1 and H2. Cognitive reappraisal was positively linked to both mental toughness (*β* = 0.162, *p* < 0.05) and distress tolerance (*β* = 0.311, *p* < 0.01), confirming H3 and H4. Finally, mental toughness demonstrated a strong positive relationship with distress tolerance (*β* = 0.559, *p* < 0.001), validating H5.

Table 4 displays the results of a mediation analysis conducted with 5000 bootstrap resamples and 95% bias-corrected confidence intervals. The analysis identified a significant indirect effect of mindfulness on distress tolerance through cognitive reappraisal and mental toughness. This effect was quantified at 0.427 (SE = 0.055, 95% CI [0.318, 0.531], *p* < 0.001), providing strong empirical support for H6.

## 5. Discussion

### 5.1. Summary of the Key Findings

This study offers a comprehensive analysis of athletes’ distress tolerance, introducing novel theoretical perspectives from various dimensions. The key findings highlight three main aspects: (1) athletes’ distress tolerance is associated not only with physical pain but also with psychological challenges such as pressure, anxiety, and anger; (2) mindfulness, cognitive reappraisal, and mental toughness are interrelated, with mindfulness having a central association with cognitive reappraisal and mental toughness; and (3) cognitive reappraisal and mental toughness mediate the association between mindfulness and distress tolerance, collectively accounting for a significant proportion of variance in distress tolerance. These findings suggest that enhancing mindfulness and emotional regulation abilities can be an effective strategy for strengthening athletes’ distress tolerance and overall performance.

### 5.2. Discussion of Each Finding

Firstly, this study highlights the dual nature of athletes’ distress tolerance, which is shaped by both physical and psychological factors. Previous research has mainly focused on the measurement of distress tolerance (Garner et al., 2018; Simons & Gaher, 2005), and its role in various behaviors (Leyro et al., 2010; Zvolensky et al., 2010) and examining its role in various behaviors (Anestis et al., 2007; Carpenter et al., 2019). Few studies, however, have explored the causes of athletes’ distress, the nature of their distress tolerance, and the impact on their athletic performance. The researchers suggest that examining athletes’ distress tolerance can help identify the root causes of their painful experiences and develop optimal strategies to enhance their tolerance. Consistent with previous research, the pain athletes experience during training and competition is the natural result of muscle strain or injuries, excessive joint use, and the accumulation of harmful metabolic byproducts in the muscles (Pettersen et al., 2020). Prolonged physical activity can alter an individual’s perception of pain, and athletes with specialized training have a higher tolerance for pain and higher pain thresholds.

However, this study extends previous findings by demonstrating that athletes’ painful experiences are not limited to physical discomfort but also include significant psychological distress. Pressure, anxiety, anger, and other negative emotions exert a continuous negative impact on athletes’ psychological states. As a physiological warning system, pain alerts individuals to potential harm and plays a vital role in minimizing exposure to damaging stimuli (Woolf, 2010). For athletes, encountering various forms of pain during training and competition is unavoidable. Despite these warnings, athletes often push their limits and take risks to secure critical points and achieve victory (Assa et al., 2019). However, in the process of overcoming physical discomfort and pushing their bodies to the limit to achieve victory, athletes also face significant psychological challenges, including the pressure of competition, public scrutiny and criticism, a monotonous and demanding training environment, and frustration and anger after losses (Gulliver et al., 2015). Additionally, sport-specific psychological issues, such as concussion-related symptoms from collisions (Grady, 2010), exercise-induced anorexia (Sudi et al., 2004), and compulsive exercise behaviors (Li et al., 2024), further exacerbate these difficulties (McNarry et al., 2020). Athletes are frequently viewed as exceptionally resilient and physically robust, which often perpetuates stigma regarding seeking mental health support (Åkesdotter et al., 2020). If left unaddressed, these challenges can escalate into greater psychological distress, potentially resulting in the loss of starting positions or contracts, which only amplifies their struggles (Bauman, 2016). If athletes are unable to tolerate their discomfort, extreme behaviors such as suicide or self-harm may occur, leading to the premature end of their careers (Baum, 2005; Rice et al., 2016).

Secondly, this study examined the interconnections among mindfulness, cognitive reappraisal, and mental toughness, as well as the direct relationship between cognitive reappraisal and mental toughness. The findings revealed significant positive correlations between mindfulness and cognitive reappraisal, mindfulness and mental toughness, and cognitive reappraisal and mental toughness (see Figure 2), aligning with the conclusions of Hill and Updegraff (2012), and Andersen (2012). Among these relationships, the impact of mindfulness on cognitive reappraisal and the influence of mental toughness on athletes’ distress tolerance are the most significant. Mindfulness meditation practice enables individuals to face the present pressure and anxiety in a non-judgmental manner. In this state, individuals consciously interrupt automatic emotional reactions, allowing attention to broaden and incorporate previously overlooked details. As habitual responses change and new information is absorbed, individuals develop new perceptions of their environment and experiences, enhancing cognitive flexibility (E. L. Garland et al., 2015; E. L. Garland & Howard, 2013).

Consistent with Levy et al. (2006), this study finds a strong relationship between athletes’ mental toughness and their distress tolerance. Athletes with higher mental toughness can withstand pressure and negative emotions, break through physical limits, and maintain excellent performance in intense competition (G. Jones et al., 2002). Additionally, cognitive reappraisal and mental toughness served as mediators in the relationship between mindfulness and distress tolerance. As shown in Figure 2, these mediators collectively accounted for 54% of the variance in distress tolerance. This study provides a clear framework for understanding the link between mindfulness and distress tolerance, emphasizing the cognitive and emotional mechanisms at play, as well as the mediating roles of cognitive reappraisal and mental toughness.

Lastly, the study underscores the practical implications of these findings, particularly the role of mindfulness in improving athletes’ distress tolerance and overall well-being. Considering the positive effects of mindfulness on enhancing cognitive reappraisal ability and mental toughness (Andersen, 2012; Hill & Updegraff, 2012), as well as its indirect influence on improving athletes’ distress tolerance (Bauman, 2016), athletes should prioritize their mental well-being. By increasing their mindfulness levels and strengthening their emotional regulation abilities, athletes can better manage their emotions and mental states during training and competition, adopting a more resilient and positive mindset. When facing physical or psychological discomfort, engaging in active cognitive reappraisal to reshape their perception of current challenges can enhance their mental toughness (Birrer & Morgan, 2010; M. I. Jones & Parker, 2018). Positive cognitive reappraisal and higher mental toughness can help athletes resist the impact of stress and other negative emotions, alleviating their sense of discomfort and enabling them to perform better during competition, showcasing their athletic skills and achieving superior results (Levy et al., 2006; Wolgast et al., 2011).

This study highlights the physical and psychological challenges athletes encounter as a result of the intense demands and pressures associated with training and competition. It underscores the potential of mindfulness meditation as an effective approach to enhancing athletes’ cognitive reappraisal abilities and mental toughness. By strengthening these abilities, athletes can better withstand stressful experiences, sustain a resilient and positive mindset, and maximize their performance, even in the face of adversity during competitions.

Furthermore, this research encourages athletes to use mindfulness meditation as a means of self-reflection, and to acknowledge and accept their mental state. When experiencing negative psychological states, they should take timely steps to adjust, such as engaging in appropriate relaxation and rest, sharing their feelings with others to relieve psychological pressure, communicating with coaches to gain insights on competition strategies and post-competition adjustments, or seeking help from mental health professionals. Experiencing psychological issues should not be viewed as shameful. Identifying and addressing such issues early is crucial to prevent them from escalating and causing significant impacts on an athlete’s performance and well-being.

### 5.3. Limitations

This study has certain limitations that warrant consideration. Firstly, potential moderating and confounding variables, such as expressive suppression, self-control, and personality traits, were not included in the model. Future research could enhance and extend the model by integrating these factors to achieve a more comprehensive understanding. Secondly, the use of a cross-sectional design in this study limits the depth and generalizability of the findings. Longitudinal studies with experimental control groups are recommended to strengthen the validity and causal interpretations of future research. Thirdly, the sampling approach, which combined snowball and convenience methods, restricted data collection to specific regions, including Hunan, Hubei, and Sichuan. This regional focus limits the generalizability of the results. Expanding the sampling to include a more diverse, nationwide population is essential for broader applicability. Lastly, the participant pool primarily consisted of athletes from a narrow selection of sports, such as badminton and athletics, leading to uneven representation. Future research should strive to include a more diverse range of athletes across various sports disciplines to enhance the comprehensiveness and credibility of the findings.

## 6. Conclusions

Based on the proposed research objectives, this study points out that every athlete experiences various psychological or physical discomforts at different stages of their career, which in turn affects their performance and results. Athletes’ distress tolerance is related to their use of emotional regulation skills, as well as their current psychological and physical state. In addition, the findings reveal that mindfulness, cognitive reappraisal, and mental toughness are important factors influencing athletes’ distress tolerance. Mindfulness influences distress tolerance through the mediating roles of cognitive reappraisal and mental toughness.

In light of these findings, the study offers the following recommendations for athletes: First, mindfulness meditation training should be incorporated into daily skill development routines to maintain a positive mindset when facing training and competitions. In daily life, improving emotional regulation abilities and redirecting attention from present difficulties to other overlooked details can foster new perceptions of challenges. During training, athletes should actively employ emotional regulation strategies, such as cognitive reappraisal, and persist through monotonous daily exercises while controlling impulsive behaviors, thus maintaining a resilient mindset and optimal performance state.

Additionally, after each competition, athletes should avoid becoming absorbed in feelings of joy or regret. Instead, they should engage in self-reflection, objectively analyze the reasons behind success or failure, and rebuild confidence to become more resilient. A more resilient mindset will enable athletes to commit to skill training and rehabilitation treatments, reducing their focus on discomforting factors and promoting better recovery and training outcomes. Lastly, athletes should not feel ashamed of their inability to tolerate pain and discomfort. When the level of pain or discomfort exceeds their capacity for regulation, they should seek professional assistance promptly to safeguard their physical and mental health.

## Figures and Tables

**Table 1:** Demographic characteristics (*n* = 285).

		*n*	%
Gender	Male	147	51.6
	Female	138	48.4
Age	Under 17	33	11.6
	16–17	83	29.1
	20–23	119	41.8
	24–25	50	17.5
Sport	Football	19	6.7
	Basketball	28	9.8
	Badminton	40	14.0
	Volleyball	27	9.5
	Table Tennis	31	10.9
	Tennis	25	8.8
	Athletics	56	19.6
	Gymnastics	12	4.2
	Taekwondo	19	6.7
	Others	28	9.8
Number of Injuries Sustained in Competitions or Training Over the Past Year	1–3	45	15.8
	4–6	67	23.5
	7–9	145	50.9
	10 or more	28	9.8

Note: *n* = total sample.

**Table 2:** Reliability and validity.

Item	Factor Loadings	Cronbach’s α	CR	AVE
Mindfulness (MI)		0.930	0.930	0.728
MI1	0.871			
MI2	0.800			
MI3	0.887			
MI4	0.872			
MI5	0.833			
Cognitive Reappraisal (CR)		0.911	0.911	0.633
CR1	0.824			
CR2	0.801			
CR3	0.862			
CR4	0.720			
CR5	0.859			
CR6	0.690			
Mental Toughness (MT)		0.914	0.916	0.609
MT1	0.725			
MT2	0.770			
MT3	0.852			
MT4	0.765			
MT5	0.850			
MT6	0.745			
MT7	0.745			
Distress Tolerance (DT)		0.886	0.888	0.666
DT1	0.808			
DT2	0.826			
DT3	0.858			
DT4	0.769			

Note: CR = composite reliability; AVE = average variance extracted.

**Table 3:** Pearson correlation.

Construct	MI	CR	MT	DT
MI	(0.853)			
CR	0.468 **	(0.796)		
MT	0.433 **	0.329 **	(0.780)	
DT	0.364 **	0.497 **	0.604 **	(0.816)

Note: The square root of the AVE is in diagonals; off diagonals are a Person’s corrections of contracts. ** *p* < 0.01; MI = mindfulness; CR = cognitive reappraisal; MT = mental toughness; DT = distress tolerance.

**Table 4:** Standardized indirect effect.

	Point Estimate	Product of Coefficients		Bootstrapping		
				Bias-Corrected 95% CI		Two-Tailed Significance
		SE	Z	Lower	Upper	
MI → DT	0.427	0.055	7.764	0.318	0.531	*p* < 0.001

Note: MI = mindfulness; DT = distress tolerance; SE = standard error; Z = Z-statistic.

## Data Availability

The data used to support the findings of this study are available from the corresponding author upon request.
